# Retracted avulsion of the distal semimembranosus tendon: MRI evaluation and surgical repair

**DOI:** 10.1016/j.radcr.2025.08.017

**Published:** 2025-08-29

**Authors:** Ekrem M. Ayhan, Jonathan M. Salandra, Michael J. Medvecky, Jim C. Hsu, Annie Wang, Lee D. Katz

**Affiliations:** a Department of Orthopedics and Rehabilitation, Yale School of Medicine, 333 Cedar St. New Haven, CT, 06510, USA; b Department of Radiology, Yale School of Medicine, 333 Cedar St. New Haven, CT, 06510, USA; c Frank H. Netter MD School of Medicine, Quinnipiac University, 370 Bassett Rd. North Haven, CT, 06473, USA

**Keywords:** Semimembranosus tear, Semimembranosus repair, Distal hamstring injuries, MRI evaluation

## Abstract

Hamstring injuries constitute up to 30% of lower-extremity pathology and are the leading cause of time lost from competition in elite athletes. Although hamstring injuries frequently involve the proximal or mid-substance regions, distal injuries are particularly uncommon and are therefore sparsely studied. Among these, biceps femoris tears predominate, whereas those of the semimembranosus and semitendinosus are less common. Distal hamstring injuries, particularly of the semimembranosus, are typically associated with concomitant meniscal, cruciate ligament, or collateral ligament injuries. We describe the rare presentation of a retracted avulsion of the distal insertion of the semimembranosus tendon, and the subsequent operative repair.

## Graphical Abstract

**Figure:**
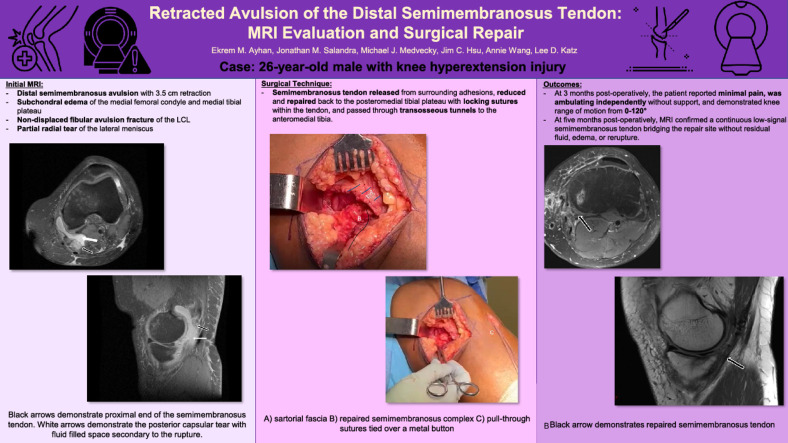

## Introduction

Among all lower extremity pathology, hamstring injuries have been reported to be as high as 30% and are the most common reason for an elite athlete to miss time from sport [1,2]. Distal hamstring injuries are particularly uncommon and have had minimal prior investigation. Of the distal hamstrings, the biceps femoris is more commonly injured than the semimembranosus and semitendinosus [3]. Distal hamstring injuries, particularly of the semimembranosus tendon, typically do not occur in isolation, as concomitant tears of the menisci, cruciate ligaments and collateral ligaments are common [4].

The hamstrings are a group of four muscles—semitendinosus, semimembranosus, and biceps femoris (short and long heads)—that primarily function in hip extension and knee flexion. These muscles follow distinct paths distally and insert at various points on the tibia and fibula. The semimembranosus, located medially and deepest among the hamstrings, has a complex distal insertion around the proximal medial tibial plateau, medial joint line, and posterior capsule [5]. These attachments provide the semimembranosus with the additional role of stabilizing the posterior capsule and posterior horn of the medial meniscus, preventing them from being compressed by the posterior medial femoral condyle during knee flexion [2,6,7].

Distal semimembranosus injuries typically result from eccentric contractions when the hip is flexed, and the knee is extended. Patients often report an acute, sharp pain in the posterior knee, sometimes accompanied by an audible “pop.” On examination, findings may include a firm mass in the posterior medial knee or a palpable area of emptiness over the posterior medial proximal tibia compared to the contralateral side [8]. However, soft tissue swelling and pain may limit the capability of these injuries to be diagnosed purely based on physical examination, thereby necessitating further diagnostic imaging.

Magnetic resonance imaging (MRI) is the gold standard for diagnosing semimembranosus injuries, with increased T2-weighted signal within the complex indicating a tear [9]. Conservative management often yields poor outcomes, making operative repair the preferred approach [10]. In cases of a posteromedial tibial plateau avulsion fracture, surgical fixation with screws or sutures for smaller bone fragments is recommended [[11], [12], [13], [14]]. Although limited clinical follow-up exists for these injuries, it has been demonstrated that most patients are able to return to full activity within four months following treatment [11].

The aim of this study was to describe the presentation, management, and outcomes of an acute distal semimembranosus tear.

## Case report

A 26 year-old male sustained a hyperextension knee injury when another person jumped on his back and he felt an immediate “pop” in the posterior knee. He had limited knee range of motion (ROM) and ambulation intolerance, and sought care in the emergency room. The patient had no significant medical history or prior knee injuries. On examination, there was diffuse knee swelling and tenderness along both the medial and lateral joint lines. Knee ROM was restricted from 5 to 20 degrees due to pain, and instability tests were inconclusive due to guarding. Anterior-posterior (AP) and lateral knee x-rays at the time showed no acute fracture or dislocation. An MRI was ordered to evaluate the injury further.

**Fig. 1:**
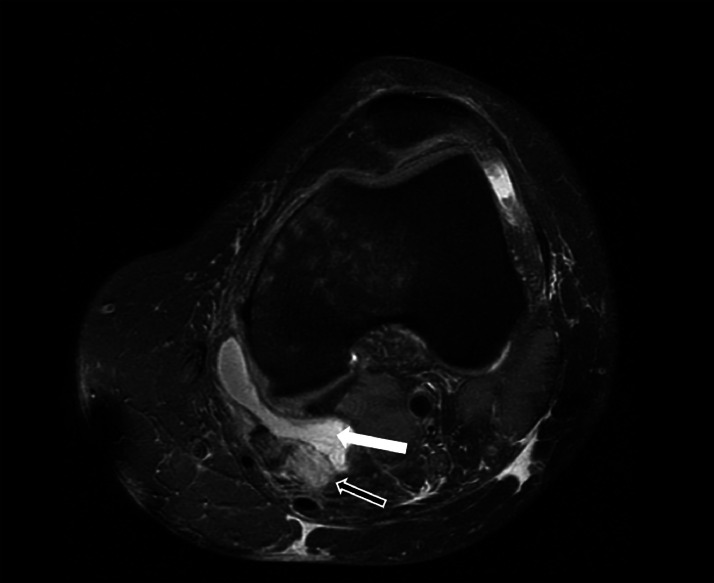
T2-weighted axial image at presentation. The black arrow demonstrates the retracted distal end of the semimembranosus tendon. The white arrow demonstrates the fluid filled space secondary to the rupture.

**Fig. 2:**
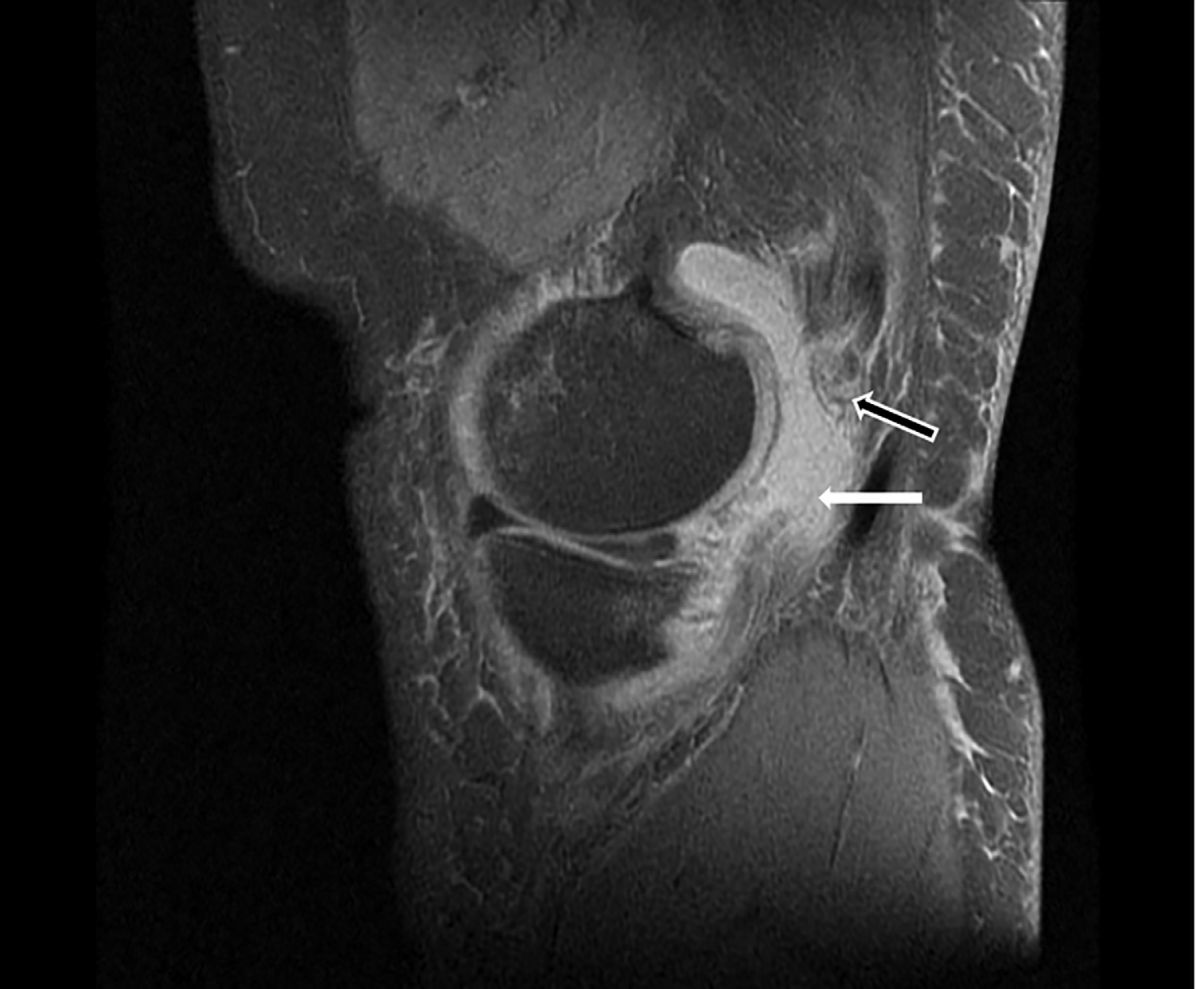
T2-weighted sagittal image at presentation. The black arrow demonstrates the retracted distal end of the semimembranosus tendon. The white arrow demonstrates the posterior capsular tear with fluid filled space secondary to the rupture.

The knee MRI revealed a distal avulsion of the semimembranosus tendon with a 3.5 cm retraction (Figs. 1 and 2), subchondral edema of the medial femoral condyle and medial tibial plateau, a non-displaced fibular avulsion fracture of the LCL, and a partial radial tear of the lateral meniscus. Following a discussion with the patient regarding the significant displacement of the semimembranosus tendon and the non-displaced nature of the LCL avulsion fracture, a decision was made to proceed with surgical repair of the semimembranosus tear and manage the LCL avulsion non-operatively.

With the patient under general anesthesia in the operating room, exam of the knee demonstrated no increased lateral joint space opening with varus stress at 0 and 30 degrees, suggesting a stable lateral ligamentous injury, consistent with his non-displaced LCL avulsion. Lachman test and posterior drawer test were symmetric to the contralateral knee, indicating intact cruciate ligaments, consistent with the findings on MRI.

**Fig. 3:**
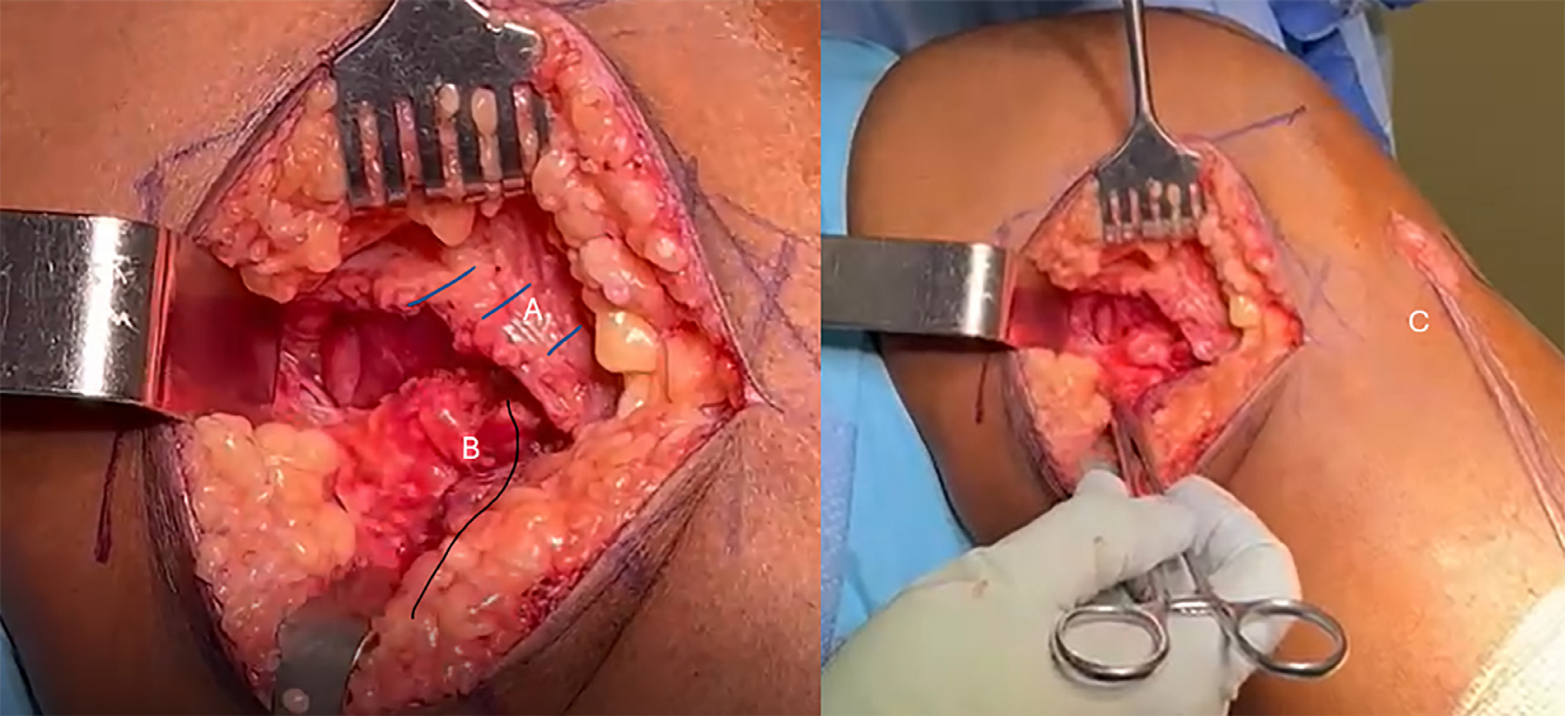
Intraoperative photograph of the repair demonstrating (A) the sartorial fascia, (B) the repaired semimembranosus complex, (C) the pull through sutures tied over a metal button.

A 10 cm incision was made along the posteromedial aspect of the knee about the joint line. After entering the sartorial fascia, the posterior oblique ligament and superficial MCL were palpated and appeared to be intact. The semimembranosus tendon was identified in a retracted position approximately 3.5 cm proximal to the joint line. The tendon was released from surrounding adhesions, reduced, and repaired back to the posteromedial tibial plateau with locking sutures within the tendon and passed through transosseous tunnels to the anteromedial tibia. The sutures were tensioned and secured over a metal button on the anteromedial tibial cortex (Fig. 3). After closure, the patient was placed in a knee immobilizer in full extension and was made touchdown weight bearing.

**Fig. 4:**
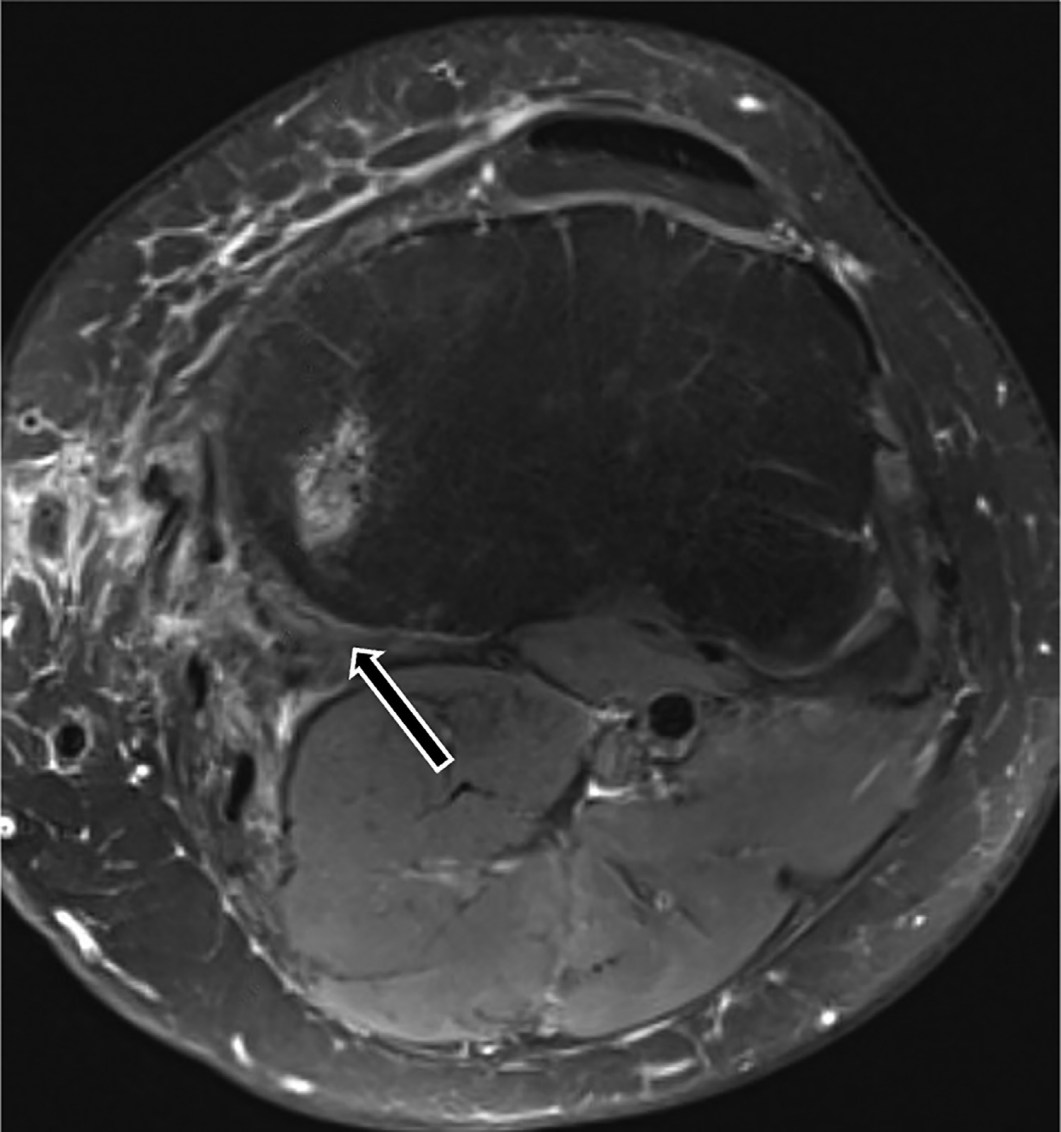
T2-weighted axial image 5 months post-repair. The arrow demonstrates the healed semimembranosus tendon at the normal posteromedial tibial plateau.

**Fig. 5:**
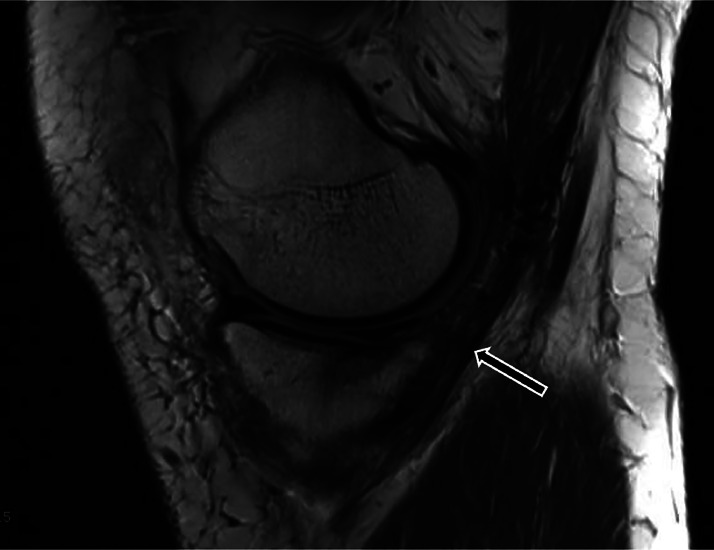
T2-weighted sagittal image 5 months post-repair. The arrow demonstrates the healed semimembranosus tendon.

One week postoperatively, the patient began physical therapy focusing on quadriceps isometrics and passive knee flexion exercises to promote ROM. By 4 weeks post-operatively, the patient achieved 110 degrees of passive knee flexion, could perform a straight leg raise independently, and reported minimal pain. Hamstring passive stretching and active strengthening exercises were not permitted at this stage. At three months post-op, the patient reported minimal pain, was ambulating independently without assistive devices, and demonstrated knee ROM from 0-120 degrees. Physical therapy for progressive active hamstring strengthening exercises were initiated. At 5 months post-operatively, MRI confirmed a continuous low-signal semimembranosus tendon bridging the repair site without residual fluid, edema, or rerupture (Figs. 4 and 5).

## Discussion

The hamstring muscle groups consist of 4 structures: the semitendinosus, semimembranosus, and the biceps femoris (short and long heads). The short head of the biceps femoris originates from the linea aspira of the femur, while the other three muscles originate from the ischial tuberosity [15]. The semitendinosus lies superficial to the semimembranosus and inserts on the proximal medial tibia as part of the pes anserinus, with accessory bands extending to the medial gastrocnemius tendon. These accessory bands are routinely released during hamstring harvest for ACL reconstruction [7]. The biceps femoris tendons are the most lateral, merging distally to insert on the fibular head. The semimembranosus inserts on the posterior medial proximal tibia through 5 distinct arms: the tibial arm, direct arm, popliteal arm, capsular arm, and oblique popliteal ligament, although variations exist [16,17]. Innervation of the hamstrings is predominantly by the tibial branch of the sciatic nerve, except for the short head of the biceps femoris, which is supplied by the common peroneal branch.

The distal semimembranosus tendon plays a dual role in facilitating knee flexion and providing stabilization to the posteromedial knee [5]. Viera et al. identified its contribution to ramp lesion development through its attachment to the posterior horn of the medial meniscus [6]. Beltran et al. utilized MRI to outline three primary functions of the semimembranosus: stabilizing the posterior capsule via the oblique popliteal ligament, synergizing with the popliteus tendon through fibrous extensions, and dynamically stabilizing the posterior horn of the medial meniscus during knee flexion, preventing excessive compression [2]. Additionally, the semimembranosus-gastrocnemius interface forms a bursa, and partial tears in the posteromedial capsule can result in a fluid collection in this bursa commonly named “Baker’s cysts.”

The hamstrings are the most commonly injured soft-tissue structures in professional athletes and typically occur in the mid-muscle belly [7]. These injuries typically occur during rapid eccentric contractions in sports such as soccer, football, and track and field. Hamstring muscle strains are the most frequent and are generally treated nonoperatively, with healing often taking up to 6 weeks. Operative repair is typically reserved for proximal ruptures involving 2 or 3-tendon ischial tuberosity avulsions [18,19].

Distal hamstring injuries, particularly those involving the semimembranosus, are rare. Mechanisms such as an axial load combined with valgus force on a hyperextended, externally rotated knee may result in semimembranosus tears, often in conjunction with posterolateral corner injuries. While partial tears may respond to nonoperative management, complete tears are associated with poor outcomes and muscle atrophy when left untreated [1,20]. Surgical repair is therefore recommended, using either suture anchors or transosseous sutures secured with a button. Alioto et al. [21] and Blakeney et al. [12] reported successful distal semimembranosus repairs in professional athletes using suture anchors. Aldebeyan et al. [22] described operative repair of a combined semimembranosus, LCL, and biceps femoris injury in a football player, who returned to play the season following his injury. Al-Humadi et al. [11] documented a construction worker with a semimembranosus-mediated tibial avulsion fracture treated with screw fixation, resulting in excellent functional outcomes.

Hamstring injuries affect athletes across all levels, from recreational to professional. This case report highlights the diagnosis and successful operative repair of an isolated acute semimembranosus tendon soft tissue avulsion injury. Awareness of concurrent knee pathology is crucial during initial evaluation, particularly on MRI, as certain mechanisms may result in combined injuries. Given the limited high-level evidence on surgical indications and management, with much of the guidance coming from case reports, early diagnosis and orthopedic intervention may be beneficial for patients, especially in cases involving tendon retraction.

## Data availability

Not applicable.

## Patient consent

Informed consent was obtained from the patient for publication of this case report and any accompanying images.
